# Autophagy maintains stem cells and intestinal homeostasis in Drosophila

**DOI:** 10.1038/s41598-018-23065-3

**Published:** 2018-03-15

**Authors:** Péter Nagy, Gyöngyvér O. Sándor, Gábor Juhász

**Affiliations:** 1 Department of Anatomy, Cell and Developmental Biology, Eötvös Loránd University, Pázmány s. 1/C, Budapest, H-1117 Hungary; 2 Institute of Genetics, Biological Research Centre, Hungarian Academy of Sciences, Temesvári krt. 62, Szeged, H-6726 Hungary; 3 Present Address: Cornell Institute of Host-Microbe Interactions and Disease, Department of Entomology, Cornell University, Ithaca, New York, United States of America; 4 Present Address: Department of Genetics, Cell- and Immunobiology, Semmelweis University, Nagyvárad tér 4, Budapest, H-1089 Hungary

## Abstract

Intestinal homeostasis is maintained by tightly controlled proliferation and differentiation of tissue-resident multipotent stem cells during aging and regeneration, which ensures organismal adaptation. Here we show that autophagy is required in Drosophila intestinal stem cells to sustain proliferation, and preserves the stem cell pool. Autophagy-deficient stem cells show elevated DNA damage and cell cycle arrest during aging, and are frequently eliminated via JNK-mediated apoptosis. Interestingly, loss of Chk2, a DNA damage-activated kinase that arrests the cell cycle and promotes DNA repair and apoptosis, leads to uncontrolled proliferation of intestinal stem cells regardless of their autophagy status. Chk2 accumulates in the nuclei of autophagy-deficient stem cells, raising the possibility that its activation may contribute to the effects of autophagy inhibition in intestinal stem cells. Our study reveals the crucial role of autophagy in preserving proper stem cell function for the continuous renewal of the intestinal epithelium in Drosophila.

## Introduction

The gastrointestinal (GI) tract of metazoans is a compartmentalized tissue with distinct specialized epithelial functions, which ensure homeostasis not only via absorption of nutrients but also by acting as a barrier. This complex tissue is generated by different populations of stem cells through spatio-temporally controlled proliferation and differentiation. Defects in these regulatory pathways may contribute to disease development and progression affecting the GI tract.

The epithelium of the Drosophila GI tract is a pseudostratified monolayer morphologically subdivided into different regions. Midgut is the most well-characterized, containing different subregions based on different morphological and histological properties, and gene expression profiles^1,2^. Multipotent intestinal stem cells (ISCs) show the highest proliferation rate in the posterior midgut (PMG), express the Notch ligand Delta (Dl), and continuously generate bi-potent enteroblasts (EBs). While ISCs and EBs both express the transcription factor escargot (esg), Notch receptor activation in EBs leads to suppressor-of-hairless/Su(H) activation and differentiation into absorptive enterocytes (ECs) expressing the POU domain transcription factor Pdm1^3^. Importantly, a subset of Su(H)^+^ EBs differentiate into class II enteroendocrine cells (EEs) expressing Prospero and specific neuropeptides (such as tachykinin and diuretic hormone 31). Prospero^+^ EEs could be also generated from Su(H)^−^ EBs (known as class I EEs), or from a specific subpopulation of ISCs expressing Prospero, indicating that EE commitment already happens at this stage^4–8^. Under both homeostatic and stressed conditions, ISC division and differentiation is controlled by several pathways such as JNK^9^, Egfr/Ras/MAPK^10–12^, Notch^6,7^, Wnt^13^, JAK/STAT^14^ and mTOR^15^. The mTOR pathway is a well-known master regulator of autophagy^16^ but its downstream effectors are unknown in the context of ISC division/differentiation.

During the main pathway of autophagy, superfluous or damaged constituents of the cell are captured into double-membrane autophagosomes, which subsequently fuse with lysosomes to ensure degradation and recycling of cargo. Pioneering studies carried out in yeast in the 1990’s identified a conserved set of core autophagy (Atg) genes, whose protein products are required for the biogenesis of the initial structures (called phagophores) and autophagosomes^17,18^. Initiation of autophagy is tightly controlled by the Atg1 kinase complex (consisting of Atg1/ULK1, Atg13, FIP200 and Atg101 in animal cells), activation of which is followed by the action of an autophagy-specific class III. phosphatidyl-inositol 3-kinase complex (consisting of Atg14, Vps34, Vps15 and Beclin1/Atg6). Potential membrane sources for the phagophore may be provided by the action of Atg9 and its regulators Atg2 and Atg18. Finally, two ubiquitin-like conjugation systems are required for autophagosome formation. The sequential action of Atg7 and Atg10 achieves covalent binding of Atg12 to Atg5, which assemble into a large complex together with Atg16. The sequential actions of Atg7, Atg3 and this complex facilitates Atg8 lipidation, a necessary step to anchor Atg8 into the phagophore and autophagosome membranes through a phosphatydil-ethanolamine tail^19^.

Surveillance of intracellular material by autophagy is crucial for cellular homeostasis, protection and survival. Autophagy occurs in all eukaryotic cells to maintain adaptation and tissue regeneration by ensuring the normal turnover of macromolecules and organelles (e.g. damaged mitochondria)^20^. Loss of autophagy in terminally differentiated neurons leads to the accumulation of toxic protein aggregates, progressive neurodegeneration and shortened lifespan^21–23^. Autophagy also maintains genome integrity by protecting cells from reactive oxygen species (ROS) produced for example during mitochondrial dysfunction^24^. Moreover, as part of the antibacterial defense, intestinal autophagy cell-autonomously protects against bacteria dissemination^25^. Intestinal autophagy improves healthspan in roundworms (Caenorhabditis elegans)^26^, but its tissue- and cell-type specific roles - particularly the stem cell-specific functions - are unknown.

It is known that autophagy inhibits the apoptotic death of mesenchymal and pancreatic cancer stem cells and promotes self-renewal of normal mesenchymal, hematopoietic, dermal and epiblast stem cells^27^. Interestingly, pharmacological stimulation of autophagy increases the reprogramming efficiency of mouse embryonic fibroblasts to induced pluripotent stem cells^28^. Autophagy influences tissue stem cell function as well. Hematopoietic stem cells in old mice need autophagy to maintain their own pool and to survive energy crisis^29^. The regenerative capacity and quiescence of aging muscle stem cells is maintained by basal autophagy, and as a consequence, its deregulation leads to premature entry into an irreversible senescent state^30^.

Even though the modulation of autophagy is an emerging new approach for the treatment of various age-related disorders including cancer and neurodegeneration, surprisingly little is known about the role of autophagy in the intestine, the organ that is usually exposed first to high concentrations of orally administered drugs. We set out to study the activity and role of autophagy in intestinal stem cells using the popular animal model Drosophila, which is likely to be relevant for understanding the physiology of our own intestine.

## Results

### Stem/progenitor cell-specific autophagy ensures proper tissue architecture during aging and regeneration

**Figure 1:**
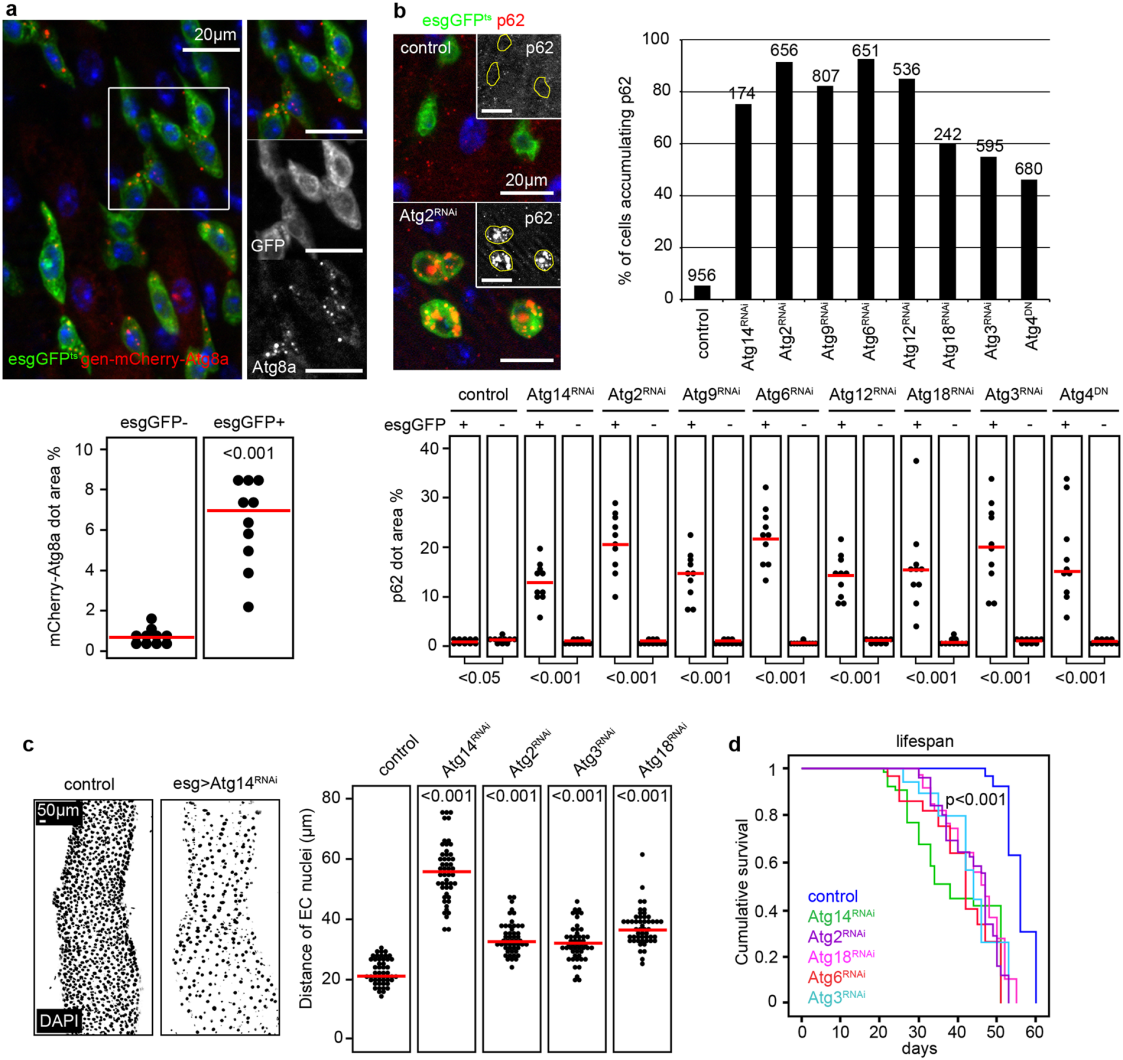
Autophagy in gut stem and progenitor cells is required to maintain tissue architecture and normal lifespan. (**a**) Esg-GFP + gut cells contain many mCherry-Atg8a autophagic structures unlike GFP-negative cells, quantified in the lower panel. (**b**) Esg+ cells accumulate p62 aggregates in response to autophagy inhibition upon Atg2/14/9/6/12/18/3^RNAi^ or overexpression of dominant-negative Atg4. Right panel shows the % of cells with large p62 aggregates, and lower panel shows the numbers of cells analyzed from 15–30 animals. (**c**,**d**) Total cell number in the PMG decreases during esg-specific block of autophagy (left panel in c), which is accompanied with increased distance between large polyploid ECs (right panel in c) and reduced lifespan (**d**). N = 10/genotype (**c**) and 92–145/genotype (**d**). P-values: two-tailed two-sample Student T-test (**a**,**b**), Kruskal-Wallis (**c**), Log-rank (**d**). Red lines: median.

To analyze autophagy in the PMG, we utilized a genomic promoter-driven mCherry-tagged Atg8a reporter to follow the endogenous activity of this degradative pathway. We observed high autophagic activity in escargot (esg)-positive stem and progenitor cells based on high number of mCherry-Atg8a dots (Fig. 1a). We also monitored the intracellular level of Ref(2)P - the fly homolog of mammalian p62, the selective autophagy receptor of ubiquitinated proteins - by immunostaining because its level negatively correlates with autophagic activity^31^. As expected in case of high autophagic activity, p62 signal was low in wild-type esg-positive progenitor cells compared to surrounding differentiated cells (Fig. 1b). Moreover, esg-specific inhibition of autophagy through Atg14, Atg2, Atg9, Atg6, Atg12, Atg18 and Atg3 RNAi or overexpression of dominant-negative Atg4 led to enormous intracellular accumulation of p62 (Fig. 1b and Supplementary Fig. S2a). Long-term oral administration of the lysosome inhibitor chloroquine blocked autophagy progression in all midgut cells, as structures positive for endogenous Atg8a or p62 accumulated after treatment (Supplementary Fig. S1). Our analysis clearly suggests that genetic modifications targeting different steps of autophagosome formation effectively suppress autophagic degradation in gut progenitor cells. Anti-ubiquitin immunostaining further confirmed these findings (Supplementary Fig. S2b). Interestingly, the distance between large polyploid EC nuclei and also progenitor cell size increased while the total cell number in PMGs decreased in response to esg-specific Atg14, Atg2, Atg3, Atg6 and Atg18 RNAi (Fig. 1c and Supplementary Fig. S2c–e). These gut tissue architecture changes were accompanied by reduced lifespan and premature death of flies (Fig. 1d).

**Figure 2:**
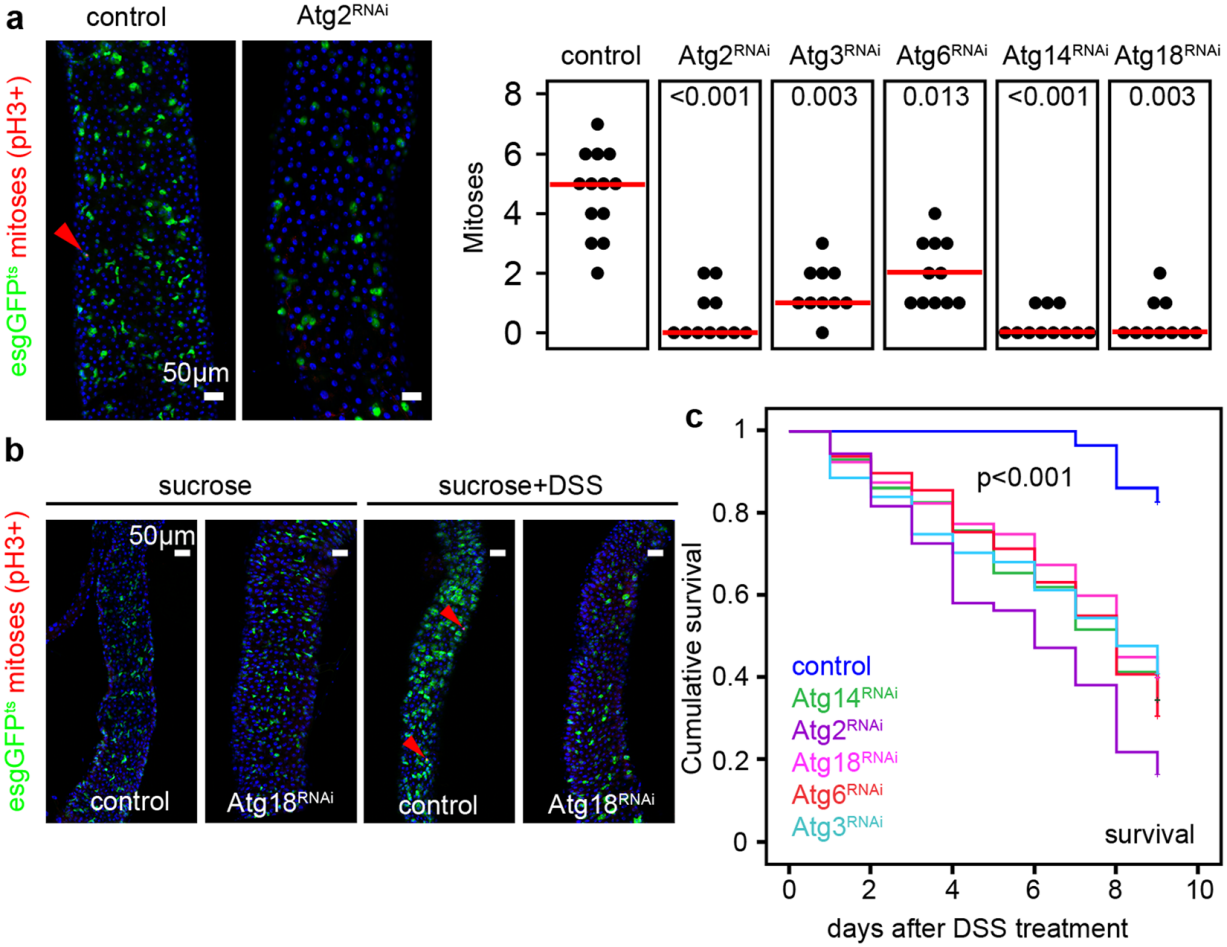
Stem cell proliferation is supported by autophagy. (**a**,**b**) Esg-specific expression of Atg2/3/6/14/18^RNAi^ inhibit stem cell mitotic activity both at the basal level (**a**), and during DSS-induced regeneration (**b**), accompanied with decreased DSS survival (**c**). Red lines: median. Red arrowheads: mitotic (anti-Phospho-histone H3/PH3 positive) cells. P values: Kruskal-Wallis (**a**) and Log-rank (**b**). N = 10–13/genotype (**a**) and 30–50/genotype (**b**).

Mitotic activity of ISCs is a characteristic response to environmental challenges and age-associated changes. Most of the esg-positive stem cells are quiescent, so normally very few mitotic cells are seen in the gut. We thus used the common anti-Phospho-histone H3 (PH3) immunostaining to identify mitotic cells. In 3-week-old animals, we observed a decrease in the number of mitoses after esg-specific inhibition of autophagy (Fig. 2a). Similarly, autophagy-deficient stem cells were unable to proliferate and produce progeny during a regenerative episode triggered by ingestion of the gut-damaging agent dextran sodium sulfate, DSS (Fig. 2b and Supplementary Fig. S3a–c), which was accompanied by decreased survival (Fig. 2c). Nevertheless, guts of genetically manipulated flies did shrink by a similar extent as control guts did upon DSS treatment (Supplementary Fig. S2d). Furthermore, the ISC/EB-specific block of autophagy increased mortality/decreased survival (Supplementary Fig. S2e,f) following oral bacterial infection with *Pseudomonas aeruginosa*.

These findings suggest that progenitor-specific autophagy is required for tissue homeostasis by enabling stem cell proliferation during aging and regeneration. Impaired ISC proliferation leads to failure to renew enterocytes that are normally turned over all the time, so the remaining enterocytes in autophagy-defective guts likely stretch out (become large and flat) in an effort to maintain gut functions as much as possible.

### JNK mediates apoptotic elimination of autophagy-defective intestinal cells

**Figure 3:**
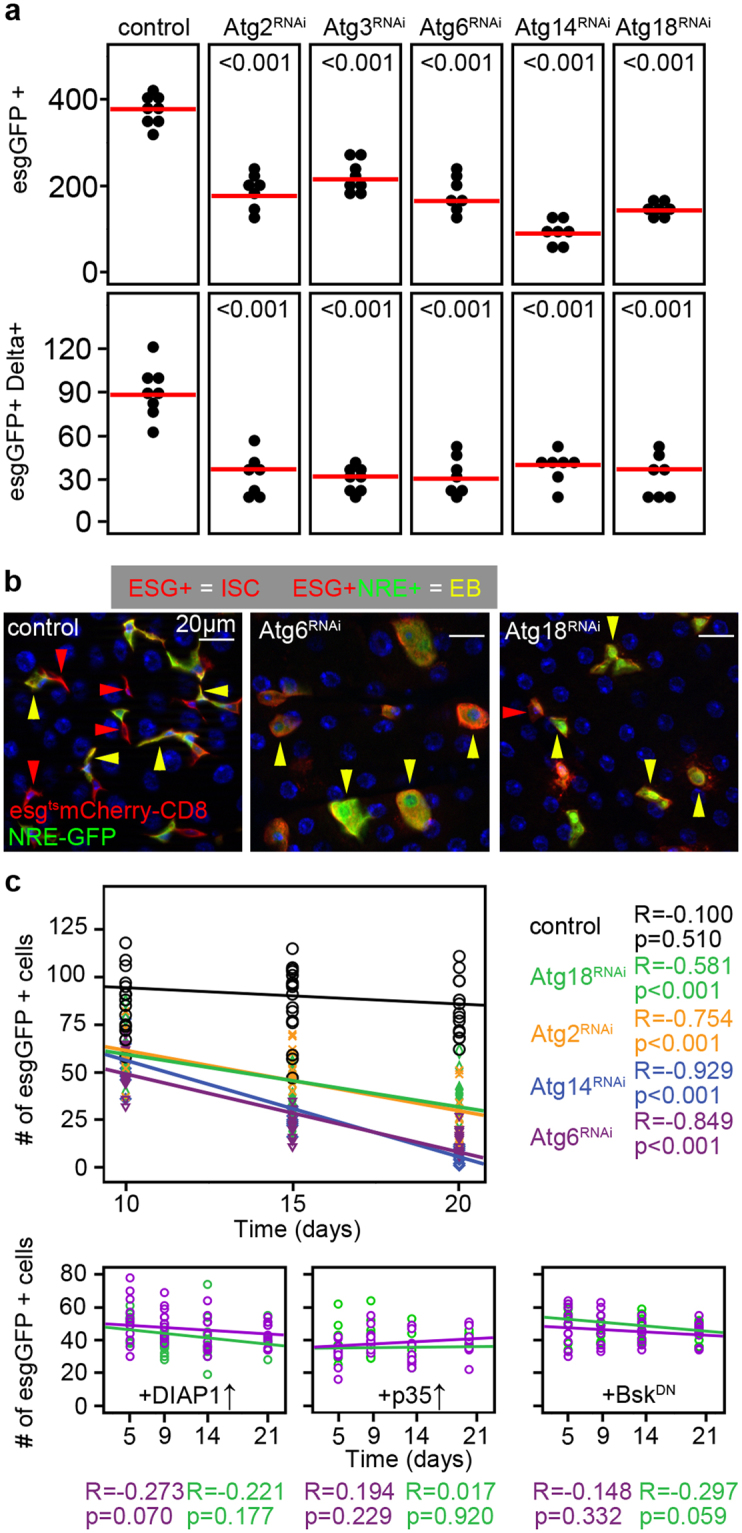
Lack of autophagy causes progressive progenitor cell elimination. (**a**) Esg-specific expression of Atg2/3/6/14/18^RNAi^ decrease the number of esgGFP +  cells and esgGFP+ Delta+ ISCs in the PMG. (**b**) The number of esg+ ISCs (red arrowheads) and esg+ NRE+ EBs (yellow arrowheads) decrease upon Atg6^RNAi^ and Atg18^RNAi^. (**c**) Linear regression analysis reveals that esgGFP + cell number negatively correlates with time during esg-specific Atg2/6/14/18^RNAi^. DIAP1, p35 and Basket^DN^ overexpression restores esgGFP+ cell numbers. Red lines: median, p values: ANOVA (**a**). R: Pearson correlation coefficient, p: significance (**c**). N = 7/genotype (**a**) and 5–10/day/genotype (**c**).

We decided to further characterize the cellular composition of guts bearing autophagy-defective esg-positive cells. Interestingly, esg-specific knockdown of different Atg genes decreased the number of esg- and Delta-positive stem cells in the PMG of 3-week old animals (Fig. 3a), and also the number of EBs positive for the Notch activity reporter NRE-GFP (Fig. 3b). Analysis of the intestine of flies null mutant for the autophagy gene Atg8a confirmed the decline of esg-positive cells in the adult intestine (Supplementary Fig. S4). Timecourse analyses of esg-positive cell numbers revealed that progenitor cell-specific Atg18, Atg2, Atg14 and Atg6 RNAi leads to progressive loss of autophagy incompetent cells (Fig. 3c). Clonal analysis confirmed the elimination of autophagy-defective cells: we observed that control and FIP200 or Atg6 null mutant clones are generated in the same ratio, but autophagy mutant cells are eliminated over time (Supplementary Fig. S5a–c). In line with this, 20-day-old mutant clones contained GFP-positive structures reminiscent of apoptotic bodies (Supplementary Fig. S5a). In addition, the total cell number within FIP200 or Atg6 mutant clones also decreased (Supplementary Fig. S5d). Interestingly, even the cellular composition of FIP200 and Atg6 null mutant mitotic clones showed several alterations compared to control: i, no Prospero-positive EE cells were seen inside clones (Supplementary Fig. S6a); ii, Delta-positive ISCs were depleted within the clones (Supplementary Fig. S6b); iii, nearly all cells within GFP-positive null mutant clones expressed the EC marker Pdm1 (Supplementary Fig. S6c).

As apoptosis and JNK-mediated stress signaling is essential for cell elimination during stress and aging^9,32^, we decided to analyze these pathways in the context of autophagy-defective cell removal. We found elevated active Caspase 3 level in progenitors after autophagy inhibition (Supplementary Fig. S7). Overexpression of DIAP1 or p35 anti-apoptotic factors or the dominant-negative form of Basket (Drosophila homologue of JNK) in Atg6 or Atg18 RNAi stem cells prevented cell elimination, suggesting that JNK mediates the stress-activated apoptotic loss of autophagy-incompetent esg-positive intestinal cells (Fig. 3c).

### Genotoxic stress induced by esg-specific loss of autophagy leads to cell cycle arrest

**Figure 4:**
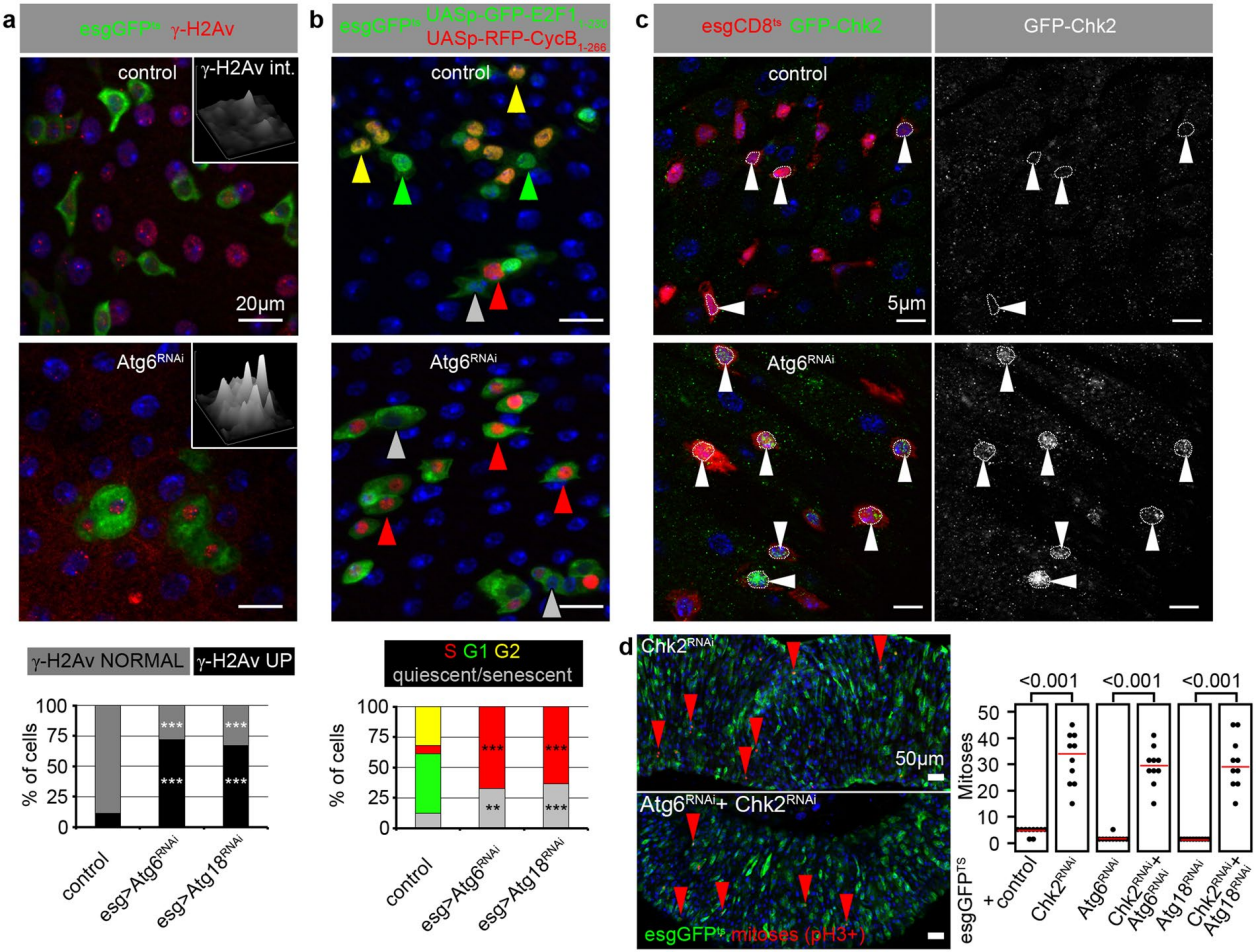
Autophagy inhibition in stem cells leads to DNA damage and cell cycle arrest, which may involve Chk2. (**a**,**b**) Esg-specific Atg6^RNAi^ elevates the level of DNA damage marker γ-H2Av (a) and attenuates cell cycle progression (**b**), quantified in the lower panels of a, b. Insets in panel a show intensity plots of γ-H2Av labeling in esgGFP-positive cells. (**c**) Nuclear GFP-Chk2 accumulation is obvious in esg+ cells expressing Atg6^RNAi^. White arrowheads point to the outlined nuclei in control and RNAi stem and progenitor cells. (**d**) Esg-specific Chk2^RNAi^ leads to ectopic ISC proliferation in both control and Atg6 knockdown stem cells. Red arrowheads: mitotic/PH3+ cells. Red lines: median. ***p < 0.001, Kruskal-Wallis tests (**a**,**b**). Red, green, yellow and gray arrowheads in Panel b indicate cells in phases S, G1, G2 or quiescence/senescence, respectively. N = 7/genotype (**a**), 11–13/genotype (**b**), 10/genotype (**c**).

Interestingly, esg-positive cells lacking autophagy exhibited elevated level of phosphorylated histone H2A variant (γ-H2Av), which marks DNA damage induced cell responses involved in cellular senescence (Fig. 4a)^33^. Senescent cells are unable to divide; accordingly, anti-Cdc2 mitotic entry kinase^15,34^ immunostainings confirmed cell division defects in stem cells lacking autophagy (Supplementary Fig. S8a). Strikingly, most of these cells expressed the S/G2/M-Green cell cycle sensor^35^ revealing that they are arrested in one of these cell-cycle phases (Supplementary Fig. S8b). To determine exactly which phase is affected, we utilized the Fly-FUCCI system to visualize cell-cycle activity during tissue homeostasis by a combinatorial expression of GFP-E2F1_1–230_ and RFP-CycB_1–266_^36^. We found that nearly 75% of autophagy-defective esg-positive cells (compared to ~7% of wild-type cells) are in S-phase and cells in G1 or G2 are nearly completely absent (Fig. 4b). Collectively, these data suggest that the block of autophagy leads to genotoxic stress and replicative stem cell senescence in the intestine during aging.

### Loss of the DNA damage-activated kinase Chk2 promotes hyperproliferation of intestinal stem cells regardless of autophagy status

DNA lesions activate Chk2 (Checkpoint kinase 2), which leads to cell cycle arrest and promotes DNA repair and apoptosis. In line with increased DNA damage in autophagy-defective cells, GFP-Chk2 accumulated in the nuclei of esg-specific Atg6 RNAi cells (Fig. 4c). Knockdown of Chk2 caused large-scale hyperproliferation of esg-positive stem and progenitor cells, and this effect was unaffected by autophagy status: Chk2 RNAi increased the number of mitoses in both autophagy-competent control and Atg6 or Atg18 RNAi cells to a similar level (Fig. 4d). In line with this, stem cell proliferation and esgGFP + cell number increased in Chk2^+/−^ heterozygous animals on control, Atg6 RNAi and Atg18 RNAi genetic backgrounds compared to control genotypes (Supplementary Fig. S9a,b). Moreover, stem cell mitotic activity increased upon esg-specific expression of dominant-negative Chk2 (Chk2^DN^), and also in Chk2^DN^ and Atg6^RNAi^ co-expressing animals compared to control genotypes. (Supplementary Fig. S9c). These data are compatible with our model that Chk2 activity may contribute to the stem cell loss seen upon inhibition of autophagy.

## Discussion

It is widely recognized that autophagy inhibition leads to tissue dysfunction, indicating a key role of this pathway in the homeostatic maintenance of post-mitotic cells^37^. Tissue homeostasis is regulated by tightly controlled division and differentiation of multipotent somatic stem cells that give rise to different cell types of a given tissue. These processes need a precisely regulated network of signals from the environment, which is translated to an adequate cellular response to ensure adaptation. Autophagy is required for mitochondrial quality control, protection from oxidative damage, energy homeostasis, quiescence, self-renewal and proliferation control in different types (such as hematopoietic, neural, mesencymal, induced pluripotent and embryonic) of stem cells^38^. In this work, we found that autophagy also plays a crucial role in sustaining proliferation capacity in ISCs, and it is required for the maintenance of the stem cell pool in the gastrointestinal tract.

Intestinal stem cells require an extraordinarily precise control between quiescent and activated states since the cellular turnover of gut tissue is the quickest in our body. It is also possible that during oral drug administration, effects on autophagy might cause defective stem cell function and intestinal hypotrophy. Sustaining the quiescent state of stem cells is crucial to ensure their reentry into the cell cycle if required. Indeed, previous results showed that satellite cells of mouse muscle tissue need autophagy to prevent senescence, maintaining stemness in this way^30^. Intestinal-specific block of Atg18 function also led to premature death and gut leakage in worms^26^. During the preparation of our work, a related study was published that found a steady-state high-level autophagy in murine intestinal stem cells, and reported that conditional Atg5 knockout increases ROS (reactive oxygen species) levels in stem cells and impairs regeneration in response to irradiation damage^39^. This is perfectly in line with our results, and illustrates that high-level baseline autophagy promotes intestinal stem cell function in various animal models under different conditions (such as radiation damage, lifespan, DSS treatment and oral bacterial infection).

Multiple additional lines of evidence indicate that autophagy is a critical effector pathway in ISCs. The central regulator of cell growth and inhibitor of autophagy is mTOR kinase. Esg-specific overexpression of the mTOR activator Rheb in flies leads to loss of gut integrity and premature death^40^, and loss of the Rheb inhibitor TSC2 also impairs ISC maintenance and gut tissue homeostasis through the mTOR pathway^15^. Although certain Atg genes may regulate ISC proliferation and differentiation independent of autophagy (such as Atg9 that directly binds TRAF family proteins to activate JNK signaling during oxidative stress^41^ and Atg16 that regulates EE maturation via Slit-Robo signaling^42^), our study demonstrates the central role of autophagy in the maintenance of ISC function during aging and stress.

Interestingly, ISC-specific loss of autophagy increases DNA damage as seen in autophagy-defective senescent mouse HSCs^43^. We found that DNA damage causes cell cycle arrest as a result of defective S-phase progression in these cells, which may ultimately lead to apoptotic elimination. Cell cycle arrest and apoptosis upon loss of autophagy may involve signaling through Chk2, a kinase activated by DNA damage to arrest the cell cycle and promote DNA repair. First, Chk2 accumulates in the nuclei of Atg6 knockdown ISCs. Second, Chk2 activation is known to arrest the cell cycle and ultimately causes apoptosis, exactly what we see upon autophagy inhibition in ISCs. Third, loss of Chk2 results in hyperproliferation of both autophagy-competent and autophagy-deficient ISCs. These observations raise the possibility that cell cycle arrest and subsequent apoptosis in autophagy-deficient ISCs is due to activation of Chk2 by excess DNA damage, likely originating from ROS production by defective mitochondria. CHK2 is a tumour suppressor gene, and its inactivation is observed in multiple types of human and mouse cancers^44^. Autophagy inhibition is currently under development as a potential anti-cancer therapy, and the lysosome inhibitor chloroquine is already used in the clinic. However, not all types of cancer cells may be susceptible for autophagy inhibition, necessitating molecular genetic analysis of individual tumours in order to select the proper treatment option. In particular, our work raises the possibility that CHK2-deficient tumour cells may not respond adequately to an autophagy inhibitor treatment.

## Methods

### Fly husbandry and treatments

Drosophila melanogaster stocks and crosses were maintained on standard cornmeal/sugar/agar medium at 22 °C, and gene/RNAi expression was induced in stem cells and enteroblasts by shifting vials containing 3–5 days old adult flies to 29 °C for inhibition of thermo-sensitive Gal80 activity, until guts were examined (typically 3-week-old adult flies unless stated otherwise). In all cases, the posterior midgut of female flies was examined. The stocks used in this study were: *esg-GFP*^*ts*^ [*esg-Gal4*, *UAS-GFP*, *Tub-Gal80*^*ts*^] (kindly provided by Philip Karpowitz)^6^; *esg-mCherry*^*ts*^ [*esg-Gal4*, *UAS-mCherry-CD8*, *Tub-Gal80*^*ts*^], *gen-mCherry-Atg8a*^45^, *w[1118]*, *UAS-Atg14 RNAi [108559KK]*, *UAS-Atg2 RNAi* [*JF02786]*, *UAS-Atg3 RNAi [101364KK]*, *UAS-Atg18 RNAi [JF02898]*, *UAS-Atg6 RNAi [JF02897]*, *UAS-Atg9 RNAi [JF02891]*, *UAS-Atg12 RNAi [JF02704]*, *UAS-Chk2 RNAi [GL00020]*, *UAS-Atg4[DN]*^31^, *NRE-GFP*^46^, *UAS-DIAP1 [BL6657]*, *UAS-p35 [5072]*, *UAS-Basket*^*DN*^, *UAS-EGFP-Mnk/Chk2*^47^, *Mnk*^*[P6]*^^47^, *UAS-EGFP-Mnk/Chk2*^*D303A*^ (*Chk2*^*DN*^)^47,48^, *UAS-GFP.E2f1.1–230*, *UAS-mRFP1.NLS.CycB.1–266*, *UAS-S/G2/M-Green*, *Atg8a*^*KG07569*^. Mutant clones were generated by somatic recombination using this MARCM stock: hsFlp; tub-Gal4, UAS-GFP; FRT82B tub-Gal80. Virgins were crossed with FRT82B Atg6[Δ1]^49^ and FRT82B FIP200^−/−^ lines. 3–5 day old mated female flies were heat shocked for 40 minutes at 37 °C. DSS treatments and oral infections were carried out as before^42^.

### Immunofluorescence, histology and microscopy

Immunolabeling of fixed guts were carried out as before^42,50^. Primary antibodies used in this study were rat anti-mCherry (1:300), rabbit anti-p62 (1:2,000)^31^, chicken anti-GFP (1:1,500, Invitrogen, A-10262), mouse anti-Delta (1:100, DSHB, C594.9B), rabbit anti-phospho-histone H3 (1:300, Millipore, 06–570), mouse anti-Prospero (1:100, DSHB, MR1A), rabbit anti-Pdm1 (1:50, gift of Fernando Jimenez Diaz-Benjumea), rabbit anti-ubiquitin (1:500, DAKO), mouse anti-H2Av (1:50, DSHB, UNC93-5.2.1), rabbit anti-Cdc2 (1:200, Santa Cruz Biotechnology, sc-53), rabbit anti-Cleaved Caspase 3 (1:50, Cell Signaling, 9661), rat anti-Atg8a (1:300)^51^ and secondary antibodies Alexa Fluor 488 anti-chicken A11039, Alexa Fluor 568 anti-rabbit A11011, Alexa Fluor 568 anti-mouse A11004 (all 1:1,500; Invitrogen). All stainings were repeated at least once, with similar results.

### Statistical analysis

All analyses were carried out as before^50,52,53^. In general, after checking for normality of data distribution, an adequate statistical test was chosen to analyze significances of differences detected between groups using SPSS 21 (IBM). Accordingly, we used two-tailed, two sample T test and Mann-Whitney U test for pairwise comparisons, and ANOVA and Kruskal-Wallis statistical tests for multiple comparisons. Hochberg and Bonferroni post-hoc tests were carried out to reduce type II errors in multiple comparisons.

## Electronic supplementary material

Supporting information
