# Supplementary material for: Autophagy maintains stem cells and intestinal homeostasis in Drosophila
Source: Sci Rep. 2018 Mar 15;8:4644. doi: 10.1038/s41598-018-23065-3 (PMC5854693; doi:10.1038/s41598-018-23065-3)
Supplement: Supplementary file 1 — Supporting information [file 41598_2018_23065_MOESM1_ESM.docx]

Title

Autophagy maintains stem cells and intestinal homeostasis in Drosophila

Péter Nagy, Gyöngyvér O. Sándor, Gábor Juhász

**Supporting Information**

**
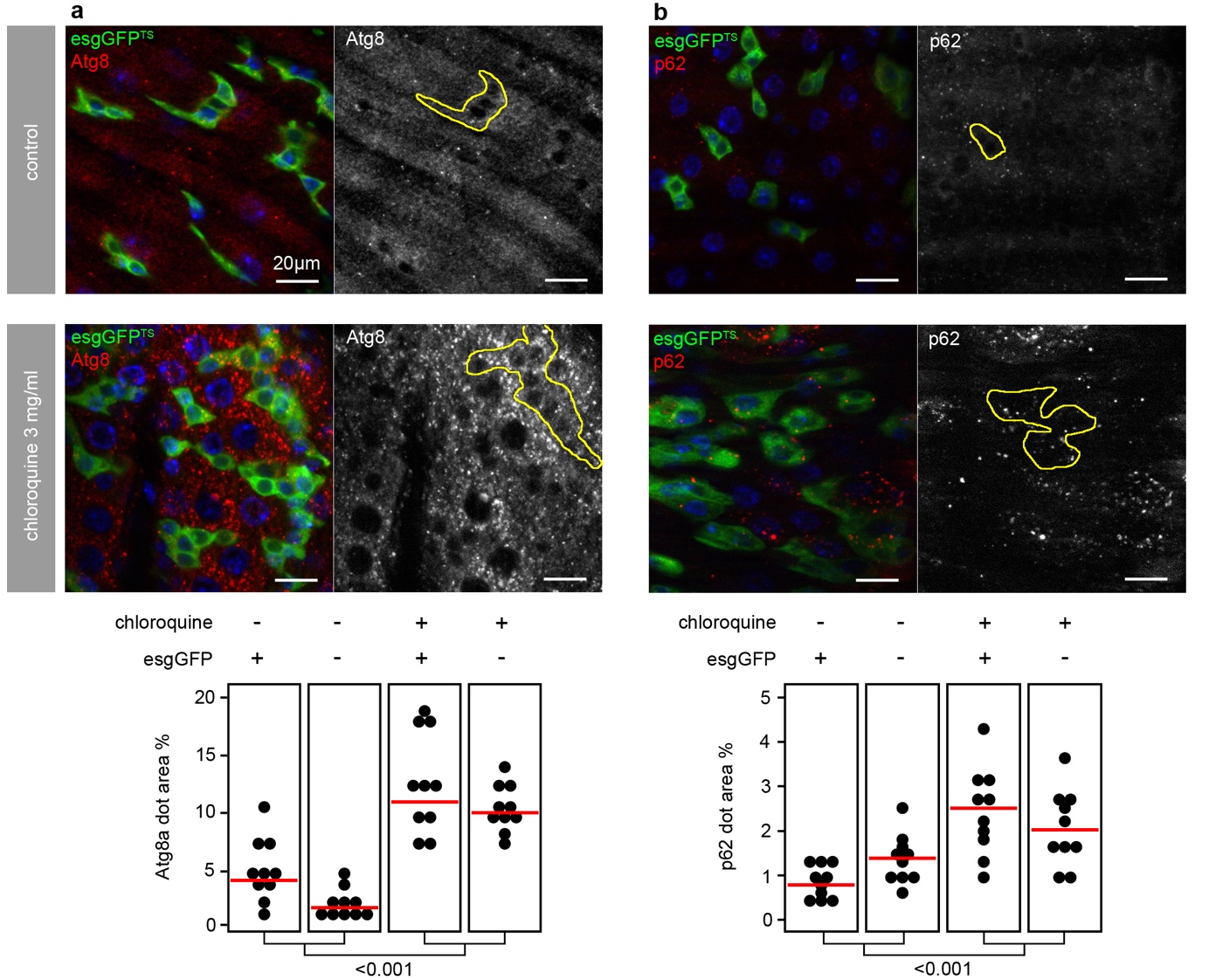
**

**Supplementary Figure S1.** Endogenous Atg8a (a) and p62 (b) accumulate in high levels in midgut cells (both in esgGFP+ or -) in response to long-term chloroquine treatment. Red lines: median, p-values are calculated from two-tailed two-sample Student T-tests for pairs of chloroquine-treated vs. non-treated animals, and N=10/genotype.


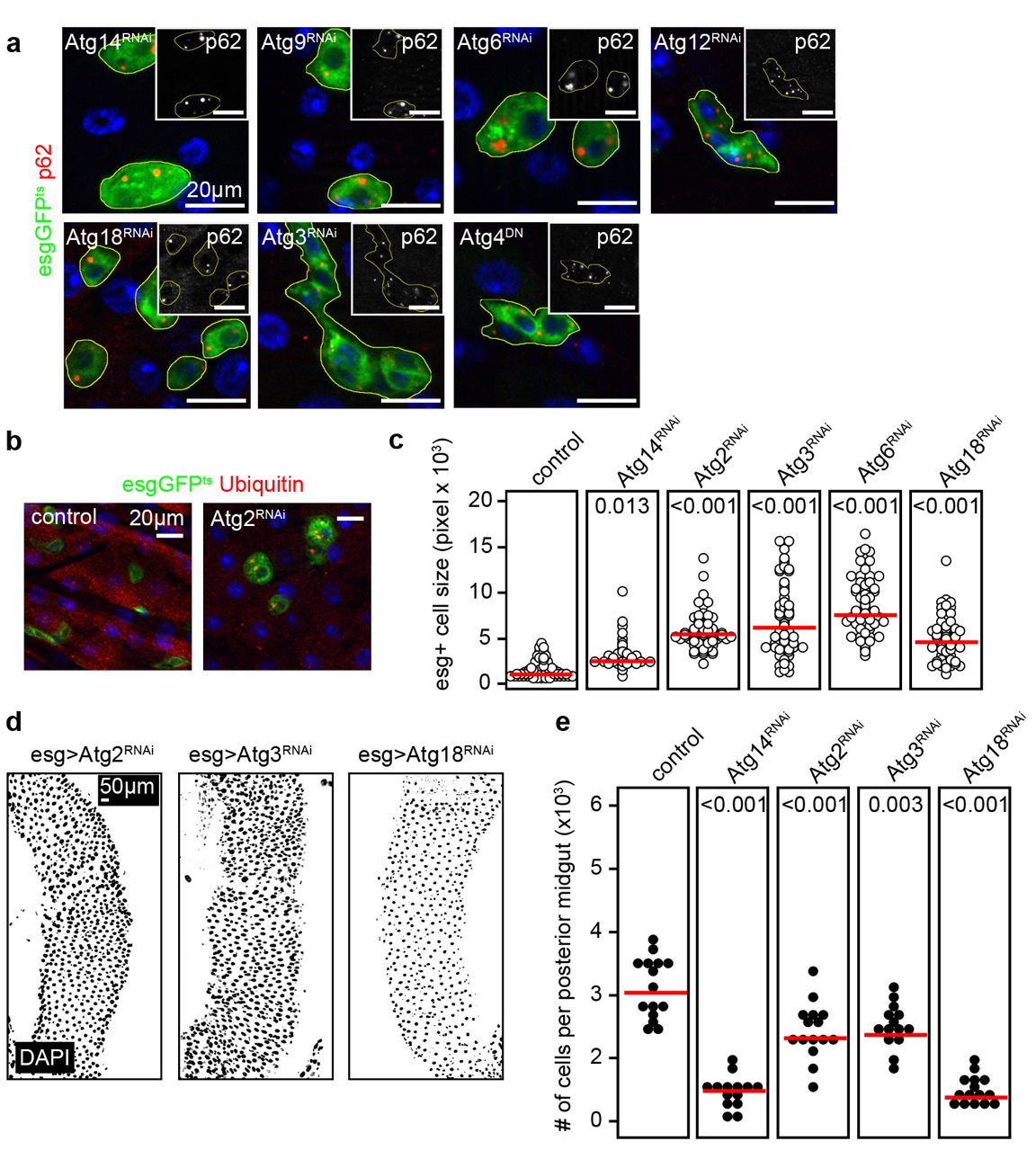


**Supplementary Figure S2.** (a) Esg-driven RNAi silencing of Atg14, Atg9, Atg6, Atg12, Atg18, Atg3 and overexpression of dominant-negative Atg4 all lead to p62 accumulation within GFP-labeled cells. (b) Ubiquitinated proteins accumulate in gut progenitors in response to esg-specific Atg2^RNAi^. (c) The size of esg-positive cells increases in response to cell-specific knockdown of Atg14/2/3/6/18. (d) Posterior midgut cell number decreases after esg-specific RNAi knockdown of Atg2, Atg3 and Atg18. (e) Numbers of DAPI-positive cell nuclei decrease upon esg-specific Atg14/2/3/18^RNAi^. Red lines: median. P-values are calculated from Kruskal-Wallis tests (c, e), and N=7-10/genotype.


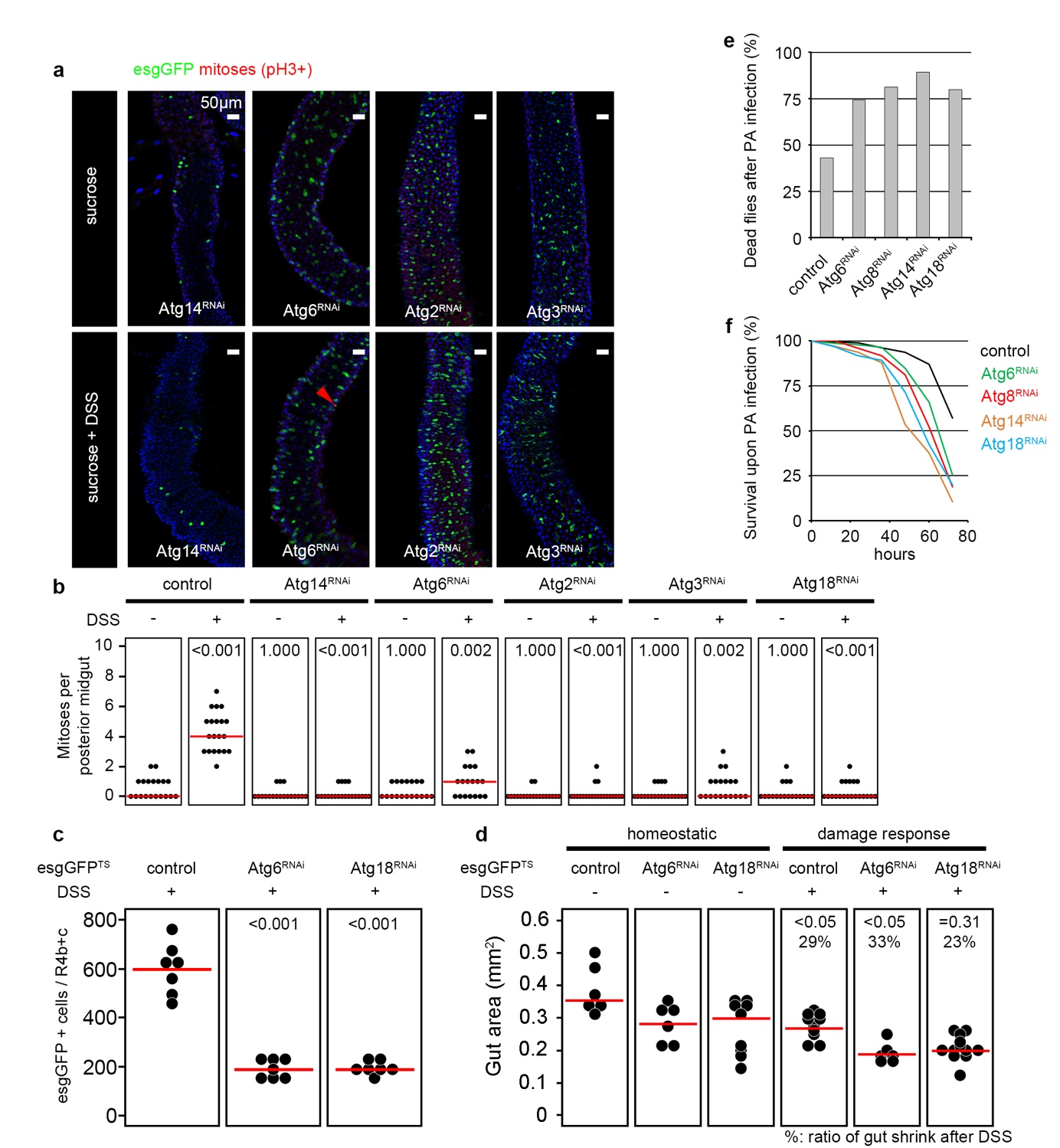


**Supplementary Figure S3.** (a) Stem cells lacking Atg14, Atg6, Atg2 and Atg3 are unable to proliferate during regeneration in response to intestinal damage by DSS treatment. Red arrowhead indicates a PH3 positive cell. (b) Quantification of mitotic nuclei after DSS treatment. P values from Kruskal-Wallis analysis of data do not indicate a statistically significant change before DSS treatment in different genotypes, while stem cell proliferation rate failed to increase during regeneration in esg-specific Atg14, Atg6, Atg2, Atg3 and Atg18 RNAi cells compared to control treated flies. Red lines: median. (c) Progenitor cell-specific Atg6 and Atg18 RNAi lowers esgGFP-positive cell numbers in the posterior midgut during a regenerative response after DSS treatment. Red lines: median. P-values: ANOVA, N=7/genotype. (d) Guts shrink by 29%, 33% and 23% after DSS treatment in control, Atg6^RNAi^ and Atg18^RNAi^ animals, respectively. Red lines: median. P-values are calculated from two-tailed two-sample Student T-tests, and N=6-12/genotype/condition. (e-f) Esg-specific knockdown of Atg6, Atg8, Atg14 and Atg18 leads to increased number of dead flies (e) and a concomitantly decreased survival (f) after oral bacterial infection of 3-week-old animals. N=18-21/genotype/condition (b) and 48-272/genotype (e, f).


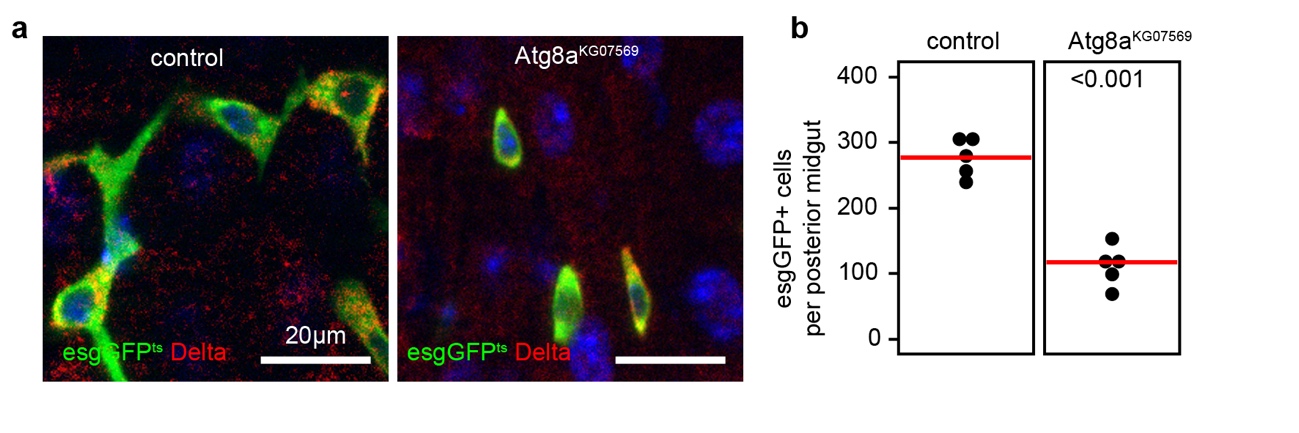


**Supplementary Figure S4.** (a-b) The number of esgGFP+ cells decreases in Atg8a mutant guts. P-value is from two-tailed two-sample Student T-test. Red lines: median, and N=5/genotype.


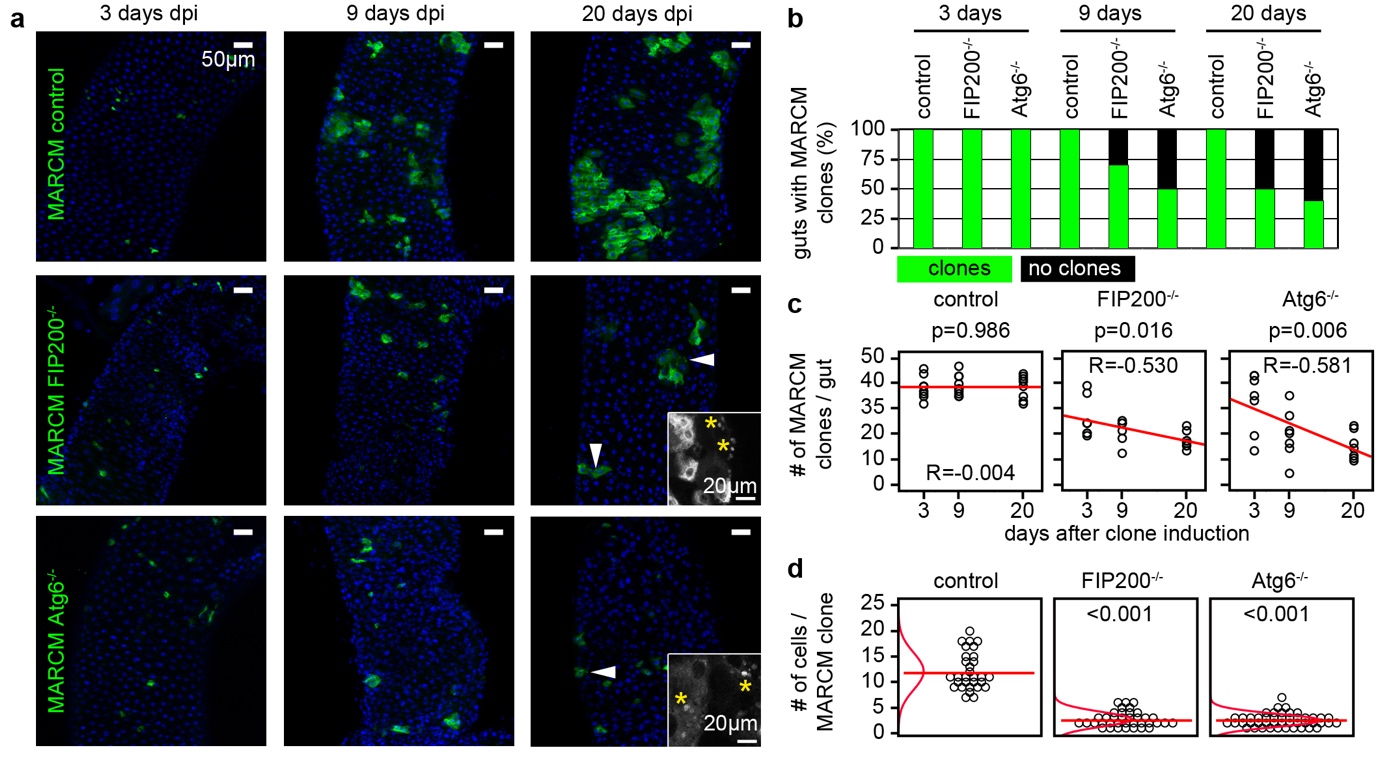


**Supplementary Figure S5.** (a) Posterior midguts containing control, FIP200^-/-^ and Atg6^-/-^ MARCM clones at 3, 9 and 20 days after induction. (b-d) The number of guts containing MARCM clones (b), the number of cells per clone (c) and the number of MARCM clones per gut (d) all decrease upon loss of FIP200 and Atg6. White arrowheads indicate cells containing putative apoptotic bodies (condensed, highly fluorescent GFP punctae), which are shown enlarged at the bottom right corner of the panels. Yellow asterisks in insets label GFP-positive intracellular bodies within MARCM clones. P values are from Kruskal-Wallis tests (d). R and p values represent Pearson correlation coefficients and significances in panel c, respectively. Red lines: linear regression showing time-dependent changes (c), or median (d). Red curves in Panel d show the distribution of data. N=27-30/genotype (a), 20-27/genotype (b), 10/genotype (d).


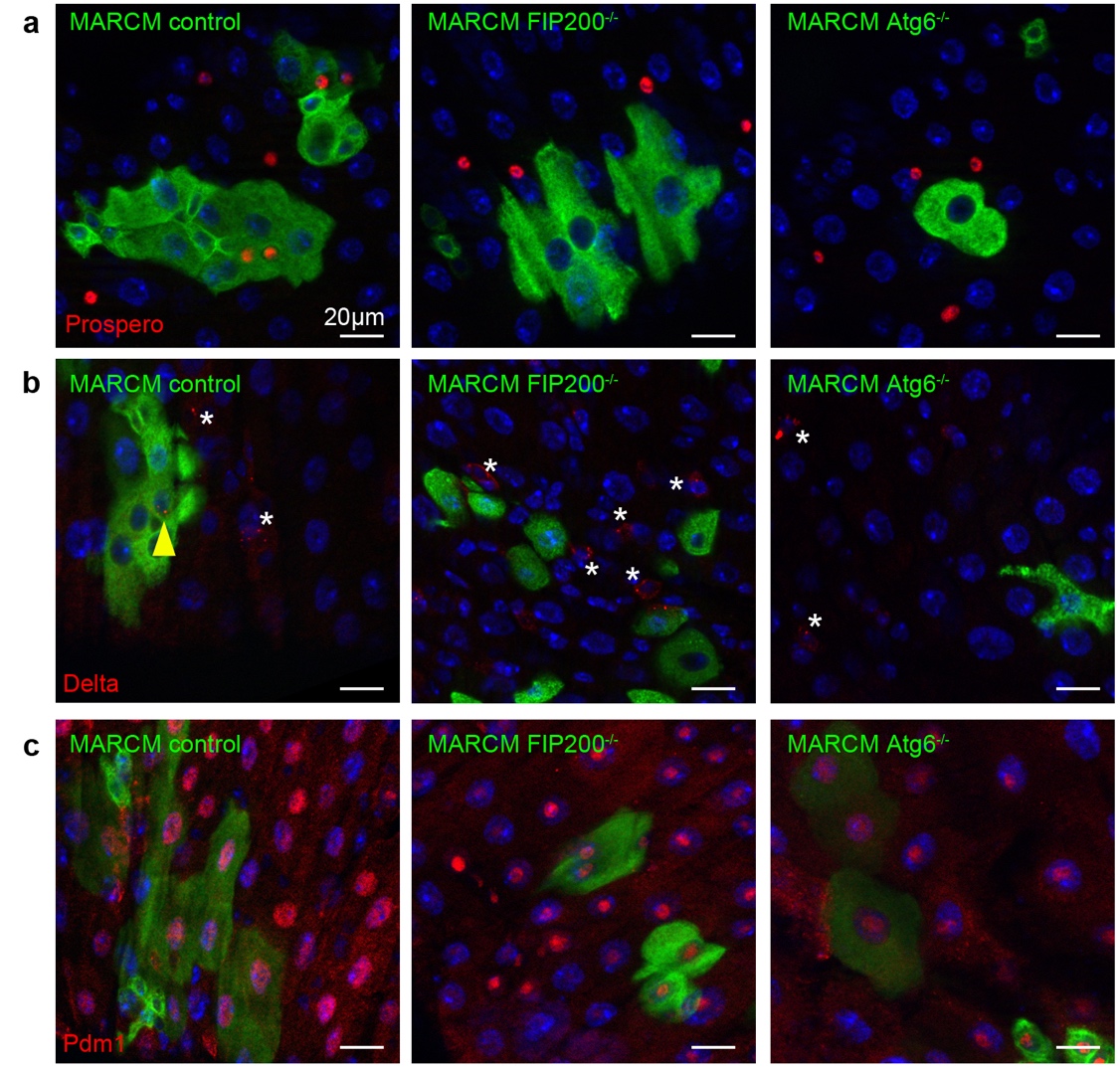


**Supplementary Figure S6.** FIP200 and Atg6 cell-autonomous mutations perturb the differentiation of Prospero+ EEs. Prospero+ cells are seen in control clones but not in FIP200- or Atg6- clones (a). Almost all cells in FIP200- and Atg6- clones are negative for the ISC marker Delta, unlike in control clones (b). White asterisks label Delta-positive stem cells outside the clones while the yellow arrowhead marks a Delta-positive cell inside the clone in panel b. Large cells seen in control and autophagy-deficient clones are positive for the EC marker Pdm1 (c).


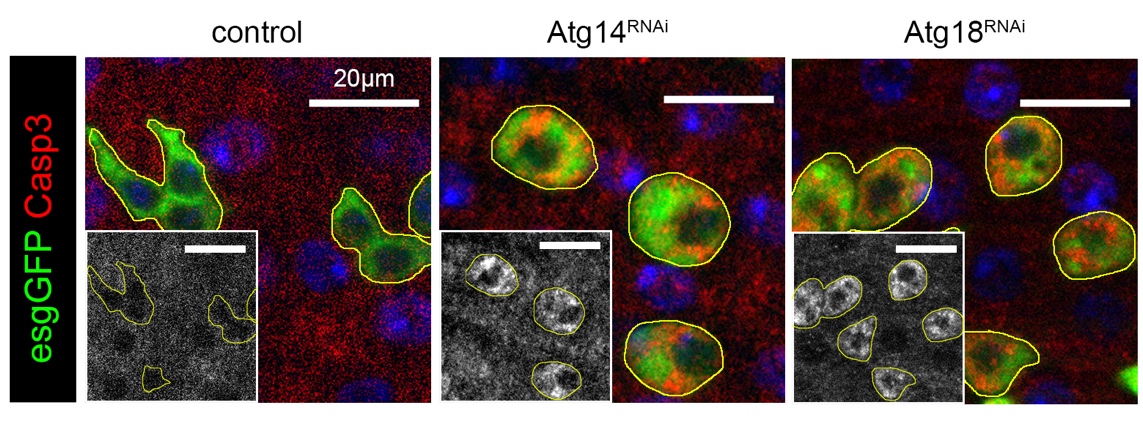


**Supplementary Figure S7.** Active Caspase-3 levels increase in progenitors after esg-specific expression of Atg14^RNAi^ or Atg18^RNAi^.


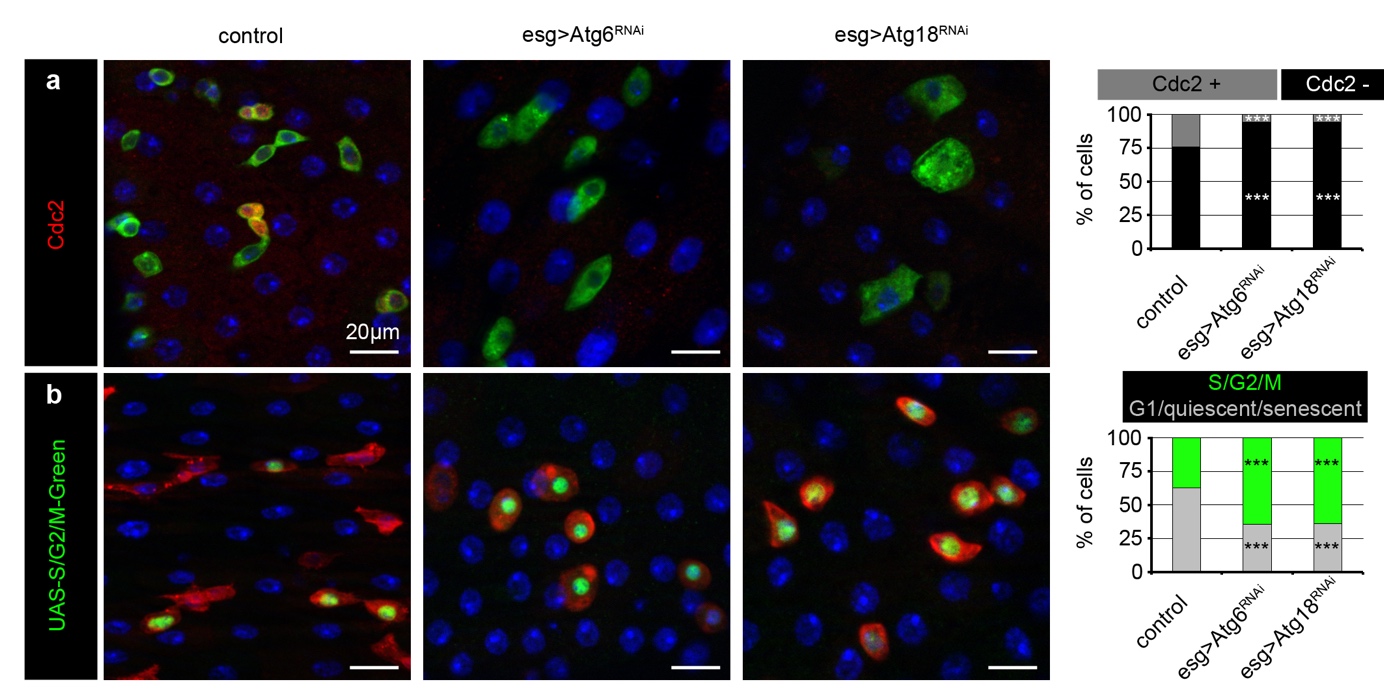


**Supplementary Figure S8.** (a) Esg-specific expression of Atg6^RNAi^ or Atg18^RNAi^ lowers the incidence of Cdc2 positivity. (b) Most autophagy-deficient cells are positive for the S/G2/M-Green cell-cycle marker. ***: p<0.001 from Kruskal-Wallis (a) and ANOVA (b) analyses, respectively. N=10-16/genotype.


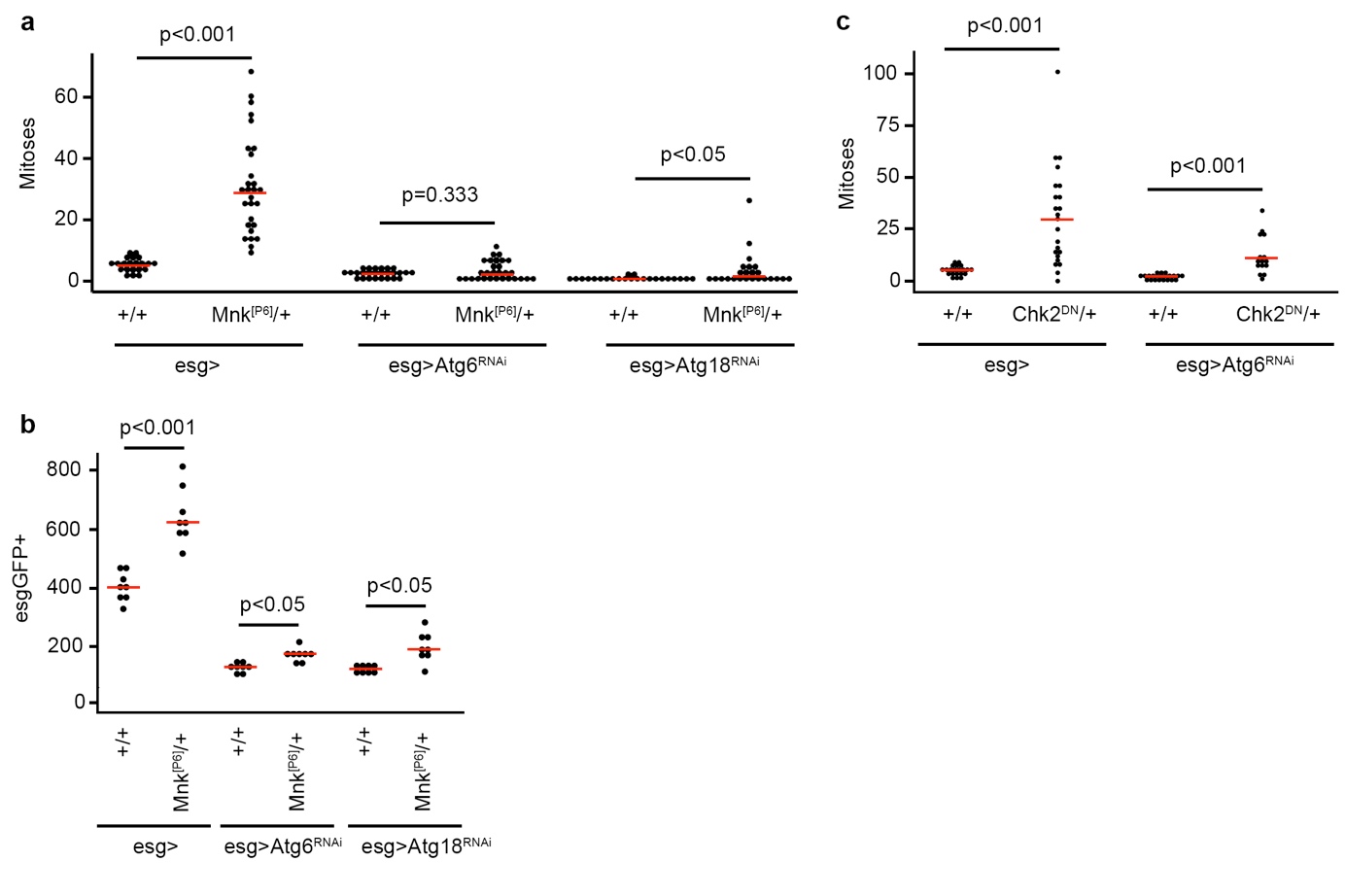


**Supplementary Figure S9.** (a-b) Mitotic index and esgGFP+ cell numbers are elevated in Mnk^[P6]^/+ heterozygous mutant flies on control, Atg6 RNAi and Atg18 RNAi genetic backgrounds compared to control genotypes. Note that all changes are statistically significant, except for the number of mitotic cells on the Atg6 RNAi background, although the same tendency is seen there. (c) Stem cell mitotic activity increases upon esg-specific expression of dominant-negative Chk2 (Chk2^DN^), and also in Chk2^DN^ and Atg6^RNAi^ co-expressing animals compared to control genotypes. Red lines: median, p-values are calculated from two-tailed two-sample Student T-tests (a-b) and Mann-Whitney U-test (c). N=8-28/genotype.
